# International Proficiency Test Targeting a Large Panel of Botulinum Neurotoxin Sero- and Subtypes in Different Matrices

**DOI:** 10.3390/toxins16110485

**Published:** 2024-11-08

**Authors:** Christine Rasetti-Escargueil, Michel Robert Popoff, Bettina Kampa, Sylvia Worbs, Maud Marechal, Daniel Guerin, Eléa Paillares, Werner Luginbühl, Emmanuel Lemichez

**Affiliations:** 1 Unité des Toxines Bactériennes, UMR CNRS 6047, Inserm U1306, Université Paris-Cité, Institut Pasteur, 25 rue du Dr Roux, 75724 Paris, France; 2 Biological Toxins, Centre for Biological Threats and Special Pathogens, Robert Koch Institute, Seestr. 10, 13353 Berlin, Germanyworbss@rki.de (S.W.); 3 ChemStat, Aarstrasse 98, CH-3005 Bern, Switzerland; info@chemstat.ch

**Keywords:** botulism, botulinum neurotoxins (BoNTs), diagnosis, proficiency test

## Abstract

Detection of botulinum neurotoxins (BoNTs) involves a combination of technical challenges that call for the execution of inter-laboratory proficiency tests (PTs) to define the performance and ease of implementation of existing diagnostic methods regarding representative BoNT toxin-types spiked in clinical, food, or environmental matrices. In the framework of the EU project EuroBioTox, we organized an international proficiency test for the detection and quantification of the clinically relevant BoNT/A, B, E, and F sero- and subtypes including concentrations as low as 0.5 ng/mL. BoNTs were spiked in serum, milk, and soil matrices. Here, we evaluate the results of 18 laboratories participating in this PT. Participants have implemented a wide array of detection methods based on functional, immunological, and mass spectrometric principles. Methods implemented in this proficiency test notably included endopeptidase assays either coupled to mass spectrometry (Endopep-MS) or enzyme-linked immunosorbent assays (Endopep-ELISA). This interlaboratory exercise pinpoints the most effective and complementary methods shared by the greatest number of participants, also highlighting the importance of combining the training of selected methods and of distributing toxin reference material to reduce the variability of quantitative data.

## 1. Introduction

Botulism is a deadly neuromuscular disease caused by botulinum neurotoxins (BoNTs). This flaccid paralysis progresses downward with clinical signs of double vision, ptosis, dyspnea, constipation, and/or nausea until paralysis of the diaphragm and pharynx provokes asphyxia [1]. Patients with respiratory distress receive ventilatory support in intensive care units and are treated with an equine botulinum antitoxin to halt the progression of the disease [2]. Thermostable spores of *Clostridium botulinum* and other BoNT-producing *Clostridium* species are present in the environment and they pose a risk of contaminating beverages or food maintained in anaerobic conditions in which they grow and release stable progenitor toxin complexes (PTCs) made of BoNT and non-toxic protein components [3,4,5]. PTCs protect BoNTs against the proteolytic and low-pH environment of the gastrointestinal tract. PTCs then dissociate spontaneously at a pH above 6.5, thereby releasing the active 150 kDa BoNT polypeptides comprised of a light chain (LC) and a heavy chain (HC) [6]. The bifunctional heavy chain contains at its carboxy-terminus the receptor binding domain H_C_ and at its amino-terminal part the translocation domain (H_N_), involved in injecting LC from synaptic vesicles into the cytoplasm of neurons. LCs are zinc-metalloprotease enzymes that cleave components of the soluble N-ethylmaleimide-sensitive factor (NSF) attachment receptor (SNARE) complex which ensures the regulated fusion of acetylcholine-containing vesicles at the presynaptic membrane of motoneurons. In cases of food-borne botulism, it is the preformed PTC that is ingested and subsequently that trigger the deadly paralysis. Despite an absence of interindividual contamination, large outbreaks due to shared consumption of contaminated food can occur [7,8]. The annual epidemiological report from the European Centre for Disease Prevention and Control reported an overall notification rate of 0.02 cases of botulism per 100,000 individuals in the population [9]. BoNTs are also typical dual-use substances with reported examples of *C. botulinum* being used in terrorist attacks and being the subject of military programs prohibited by the Biological Weapons Convention [10,11] but they are also licensed for therapy for a growing number of neuromuscular disorders and aesthetic medicines [12,13]. To fulfill the technical demands of BoNT detection and quantification, different functional, immunological, and mass spectrometry-based methods have been developed [14].

BoNTs comprise a family of more than 40 subtypes which are divided into serologically different groups (serotypes A–H). BoNTs show variations in their amino acid sequences comprised between 37.2% and 69.6%, and can be further divided into subtypes that display amino acid variations within a serotype greater than 2.6% with a few exceptions within the E serotype [15,16]. BoNT serotypes reported in Europe were predominantly serotype B. Nevertheless, botulism cases due to serotypes A, E, or F are reported regularly [9,17]. Despite the large spectrum of sequence variations reported to date, only a few neurotoxin subtypes belonging to the serotypes A, B, E, and F are commonly responsible for human botulism, and strong geographical distribution is also shown [15]. The intensification of food trading contributes to the risk of the diversification of BoNT subtypes in human botulism, thereby requiring the development of diagnostic techniques targeting the broadest spectrum of BoNT subtypes. In respect of this, the mouse bioassay (MBA) introduced in the 1920s by Bengtson is still included in official methods and national guidelines of diagnostics since it detects all known BoNT variants pathogenic to humans. To challenge the MBA, a method was set based on paralysis detection of an electrically stimulated hemidiaphragm explant, referred to as HDA or MPN [18,19]. This method has the advantage of reducing animal suffering and of providing quantitative data.

Non-functional *in vitro* methods have been developed to improve the sensitivity of BoNT detection and reduce *in vivo* experiments in compliance with the European directive 2010/63/EU. Shared epitopes within each BoNT serotype allow for the challenging generation of monoclonal antibodies suitable for immunological methods of BoNT detection. This encompasses the sandwich ELISA, which is by far the most employed *in vitro* method [14,20,21,22,23]. ELISA provides valuable information on serotypes but does not allow subtyping. Depending on the quality of antibodies, these robust methods can reach high sensitivity for multiple samples in a short period of analysis. This holds for immunochromatographic lateral flow assays, which are hand-held devices based on a sandwich ELISA performed on paper strips [14].The growing misuse of BoNT counterfeits in unregulated cosmetic applications, with a rise of iatrogenic cases, emphasizes the need to develop diagnostic tests that are sensitive enough to detect as low as 0.1–1 pg/mL of BoNT/A1 toxin in human serum [24]. Indeed, BoNTs are one of the most lethal biological substances known to humankind with lethal doses estimated to be 1 ng/kg and 1 μg/kg bodyweight for contaminations by the parenteral and oral routes, respectively [25]. To address the question of detection sensitivity, several *in vitro* methods aimed at detecting LC endopeptidase activities have been developed. This offers the advantage of detecting the fraction of toxin enzymatically active and shows a serotype-specific signature in SNARE cleavage. This group of functional *in vitro* methods implement different read-outs, including either a mass spectrometry (Endopep-MS) [26] or a immunological detection of cleavage products (Endopep-ELISA) [27,28]. The sensitivity of this type of assay is similar to or even better than MBA when coupled with the step of immunocapture [14,29]. Independent of the aforementioned *in vitro* approaches, different cell-based assays relying on the detection of cells intoxicated by BoNT have been developed [30,31]. Finally, the development of methods aimed at miniaturizing the detection of BoNT or BoNT endopeptidase activities has also been reported, and this encompass microarrays or biosensors, centrifugal microfluidic disk platforms, and portable devices [32,33,34,35].

Previous proficiency tests (PTs) conducted in the European EQUATOX program pointed out the importance of developing certified reference material for reporting normalized quantitative results [36]. Here, we included recombinantly expressed BoNT/A1 (recBoNT/A1), as the calibrant for BoNT/A quantification, in line with its certification as EU reference material EURM-111 [37]. Finally, the EuroBioTox program targeted the importance of implementing training courses to disseminate standardized methods of diagnostics among participants.

## 2. Results and Discussion

This international PT conducted in the EuroBioTox program involved a large panel of European laboratories specialized in the diagnostics of botulism as well as a large spectrum of BoNT serotypes and subtypes. Many participants were enrolled into training courses of selected immunological methods implemented in this PT exercise. This PT also included recombinant recBoNT/A1 as the calibrant for BoNT/A quantification. The scheme of this PT was set to meet internationally acceptable standards including sample stability and homogeneity testing as well as data anonymization [38]. Here, we included 16 test samples in total. This comprised 3 blank samples and 13 samples spiked with BoNT/B (8/13), and BoNT/A (4/13) subtypes, either recombinantly expressed (recBoNT) or native PTC (referred to as BoNT), as well as native BoNT/E and BoNT/F. Samples were spiked at theoretical concentrations of 0.5, 10 and 40 ng/mL. Higher concentrations were chosen to obtain results from various methods including fewer sensitive methods. The low concentration was also used to challenge the analyses and gain more insights on method sensitivities. Since the focus of EuroBioTox was more on BoNT/A and/B, low concentrations in the PT were selected for these serotypes. Finally, the neurotoxins were spiked into milk as an example of the food matrix, into soil as an example of an environmental matrix, and into human serum as a standard clinical matrix.

Here, we detail methods implemented by the participants, as well as discussing the qualitative and quantitative data with regard to methods and participants.

### 2.1. Selection of the Samples

This PT was aimed at comparing the performance of qualitative and quantitative methods of diagnostics implemented by expert laboratories to detect commonly encountered BoNTs sero- and subtypes responsible for human botulism. Native semi-purified PTC and recombinantly expressed BoNT (recBoNT) were produced and characterized, as discussed in the Materials and Methods section. BoNT subtypes that were included in this PT primarily encompassed several BoNT/B subtypes (B1, B2, and B5) and BoNT/A subtypes (A1, A3, and A4), including the mixed native BoNT/B5 and BoNT/A4 (BoNT/B5a4) produced by the bivalent strain Ba657 of *Clostridium botulinum* (Table 1). BoNT/B1 displays 4.4% and 3.9% maximum amino acid differences with B2 and B5, respectively [15]. The difference between BoNT/B2 and BoNT/B5 reaches 4.7% of maximum amino acid variation. The overall amino acid differences between subtypes among the BoNT serotype A were even greater with recorded values between 10.6% and 15.6% for BoNT/A1, A3, and A4. We also included native BoNT/E1 and BoNT/F1, as well as recombinantly expressed and fully processed BoNT/A1 (recBoNT/A1) and BoNT/B1 (recBoNT/B1). Three different matrices representative of clinical (serum), environmental (soil), and food (milk) specimens were selected to estimate possible matrix effects on the sensitivity and specificity of the methods (Table 1). One vial containing recBoNT/A1 was also included to standardize BoNT/A quantification [36]. Antibodies for the capture and detection of BoNT/A and BoNT/B serotypes were available for PT participants at the EuroBioTox repository.

Details on sample preparation are reported in the Materials and Methods section. Briefly, samples spiked with BoNTs were selected after the evaluation of BoNT stability, and sample homogeneity was carried out on randomly selected tubes, together with the determination of assigned concentrations by the organizing laboratory, e.g., concentrations of BoNTs measured by ELISA after spiking (Table 1). The theoretical concentrations of BoNT spiked in the different samples ranged from low (0.5 ng/mL) to medium (10 ng/mL) and high (40 ng/mL) concentrations. It is noteworthy that most assigned concentrations, notably for BoNT/E, gave lower values, as compared to theoretical concentrations (Table 1). This is possibly due to losses during sample preparation, matrix interference effects, or other disturbing factors. Nevertheless, as detailed in the Materials and Methods section, the stability and homogeneity of samples were established before sample dispatch. Of note, two samples, S05 (recBoNT/B1 at 10 ng/mL in serum) and S09 (BoNT/B5a4 40 ng/mL in soil), that did not meet these criteria during the preparation phase of the PT were excluded from the exercise. Finally, a second series of stability tests after completion of the PT established the adequate stability of spiked samples during the time course of the exercise.

In total, 16 samples including 3 blank samples were sent to 19 participants.

### 2.2. General Aspects of the Exercise and Reported Results

All participants were informed about the objectives of the PT including the formalism in reporting the results in a standardized data report-sheet for subsequent statistical analyses. Participants were asked to qualitatively analyze the presence or absence of BoNT in all samples with, if possible, BoNT serotyping.

Laboratories involved in the PT were asked to use at least one of the ELISA methods from training during EuroBioTox, e.g., ELISA RKI-1 (EL.R-1A) and ELISA CEA (EL.C-A). Capture and detection antibodies targeting BoNT/A and BoNT/B serotypes were made available to participants on the EuroBioTox repository in order to implement the ELISA method EL.R-1A covering all four BoNT serotypes and CEA.C covering serotypes A, B and E. Indeed, including standardized procedures, through the implementation of training courses and shared reagents allowed direct interlaboratory comparison of data.

Qualitative and quantitative measurements had to be conducted over a period of 4 weeks in independent duplicates (including all steps of pre-analytical sample preparation). Quantitative results for samples spiked with BoNT/A and BoNT/B had to be reported, except for samples S02 and S06, which were assigned for qualitative analyses only. For quantification of BoNT/A and/or BoNT/B, the participants were requested to provide their raw data for five predefined samples [S03 (blank serum), S04 (BoNT/B1), S13 (BoNT/A1), S14 (BoNT/A3), and S17 (BoNT/B1)]. This included values of absorption and standard curves, together with associated information on the protocol, such as concentrations and dilutions. For BoNT/A, data had to be calculated with at least a standard curve performed with the candidate reference material recBoNT/A1. For the mass spectrometry (MS) methods targeting BoNT/A and/or BoNT/B, the participants were requested to provide the peptide fingerprints (MS/MS data) of three predefined samples [S07 (unspiked milk), S14 (BoNT/A3), and S18 (BoNT/B2)].

Finally, as an option, participants were requested to determine BoNT subtypes, if detected, in the blank milk sample (S07) and buffer-containing samples spiked with 10 ng/mL of BoNT/A1 (S12), BoNT/A3 (S14), and BoNT/B2 (S18).

In total, only 18 participants among 19 reported data gathered from not less than 39 analytical methods listed in Table S1. This included different immunological methods with different pairs of antibodies for the capture and detection of BoNTs. The number of laboratories reporting data for each of the four BoNT serotypes varied as follows: 18 laboratories reported data for BoNT/A, 16 for BoNT/B, 15 for BoNT/E, and 11 for BoNT/F. This represented 38 datasheets for all BoNT serotypes combined. A majority of nine laboratories reported results based on one analytical method, with four laboratories reporting results with two methods, three laboratories with three methods, and two laboratories reporting results from four or more methods. The qualitative results as well as the conclusive results summarizing the data from all methods used and reported by the participants were anonymized.

**Figure 1:**
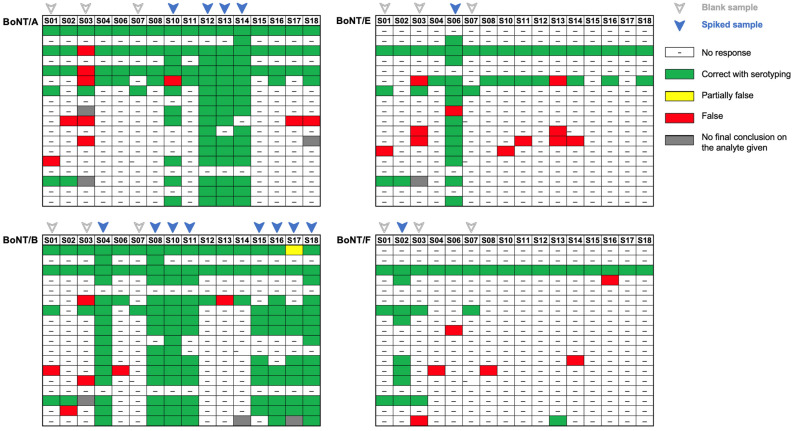
Results reported by the participants after anonymization (lines) for each BoNT and by sample described in Table 1 (columns). Green indicates that the participant reached a correct conclusion based on the results of one or more methods implemented. Red indicates a wrong conclusion. The absence of reported data is indicated as (-). For some reported data, more details are provided according to the figure legend: partially false (yellow) and no final conclusion (grey). The samples spiked with the indicated BoNT serotype are indicated with a blue arrowhead and blank samples with a grey arrowhead.

The conclusive results reported by the participants for BoNT/A, B, E and F are depicted in Figure 1 using a color code for evaluation of the reported results. This color code allows direct visualization of the correct (green) and wrong (red) conclusions reported by the participants based on results gathered with one or more methods.

The overall correct conclusions (green) provided by the participants, e.g., either the presence or the absence of each of the four BoNT serotypes, established the adequate preparedness of specialized laboratories in Europe and abroad. Reaching such a high rate of correct conclusions was all the more remarkable considering that this exercise included several BoNT serotypes and subtypes, with a mixed BoNT-containing sample, as well as complex matrices, including serum. Indeed, a large majority of participants correctly identified the presence or absence of BoNT/A, B, and E. Eight participants reported conclusions for BoNT/F in S02 that were all correct. Another striking observation based on the conclusions brought by participants of this PT is that they had more difficulties identifying the blank matrix serum (S03), as compared to soil (S01) and milk (S07), giving numerous false positive results whatever the targeted BoNT serotype A, B, or E.

The overall success of the participants in reaching conclusions on the presence or absence of the BoNT serotypes allowed for a more detailed analysis of responses by method, method principles, participants, and analytes, as discussed in the following sections.

### 2.3. Qualitative Results by Method and Groups of Methods

To analyze the data as a function of the analytical strategies, we divided the different methods into three different groups (Table S1). Methods of group I correspond to functional methods developed to detect BoNT biological activities *in vivo* and *ex vivo*, as well as BoNT enzymatic activities *in vitro* on SNARE substrates. These functional methods encompassed mouse-based experiments, e.g., the standard *in vivo* MBA, as well as the *ex vivo* MPN or HDA. It should be kept in mind that these two methods were the only ones from group I to assess the ability of active BoNTs to enter into neurons and trigger effective neuromuscular paralysis. In addition, group I methods included those based on the detection of neurotoxin endopeptidase activities, e.g., Endopep-ELISA (serotypes BoNT/A and B) and Endopep-MS. The methods classified in group II encompassed immunological methods detecting the presence of BoNT proteins. This group gathered the largest number of methods including several types of ELISAs, which differ depending on the antibodies used to capture and detect BoNT, immunochromatographic lateral flow assays (LFA), and microarrays. Data from the Liquid Chromatography-coupled to Tandem Mass Spectrometry (LC-MS/MS) method were analyzed separately in a third group (group III). Since these approaches taken together combined different analytical principles, the classification of methods should be regarded more as general guidance.

Table 2 reports qualitative data processed to highlight correct positive (presence of BoNT) or correct negative (absence of BoNT) responses by groups of method and for each BoNT serotype. Table 2 also displays the sum and details of the percentages of the results reported as correct positive and correct negative. One should keep in mind that the distribution of correct positive and negative results is highly dependent on the number of samples spiked with BoNT from a given serotype (correct positive) and blank samples (correct negative), while the total percentages translate the overall level of positive responses (both correct positive or correct negative).

Taken collectively, these data report information regarding the overall occurrence of methods implemented by the participants, reflected in the total number of results, here referred to as total count. Strikingly, when comparing total counts between the three different groups of methods, we noticed about twice as many counts reported with immunological methods than with functional methods, except for BoNT/F, for which both groups of methods gave values of total count in the same range. Note that the LC-MS/MS method was implemented by only two participants, giving low numbers of total count. We then analyzed values of total count between groups of methods and BoNT serotypes. We noticed that both groups of functional and non-functional LC-MS/MS approaches gave similar values of total count for all four BoNT serotypes. In contrast, values of total count reached with the immunological methods were about 1.5-fold less for BoNT/E and 4-fold less for BoNT/F than for BoNT/A and BoNT/B. The higher values of total count recorded for BoNT/A and BoNT/B align well with the fact that antibodies targeting BoNT/A and BoNT/B were available on the EuroBioTox repository. Strikingly, we noticed that the highest values of the total count, between methods targeting all four BoNT serotypes, were reached with Endopep-MS (from 144 to 181 total count). When comparing methods targeting one of the three serotypes of BoNTA, B and E, we found that the highest values of the total count were obtained with the lateral flow assay (LFA) CEA Multiplex (LFA.CEA-Multi), followed by EL.R-1 and Endopep-MS. Of note, two more counts were reported with EL.R-1 targeting BoNT/A than with BoNT/B or BoNT/E. Considering trained methods, we recorded about a four-fold greater value of total count with EL.R-1A than with EL.C-A. The EL.R-1B and EL.R-1E gave about two and four-fold higher values of total count than EL.C-B and EL.C-E, respectively. The EL.R-1 method targeting BoNT/F gave a lower value of total count (*n* = 32) although we recorded an excellent percentage of 96.9% of correct results.

In conclusion, the participants reported the highest number of total counts for the four BoNT serotypes with the EL.R-1 and Endopep-MS methods, as well as LFA.CEA-Multi for BoNT of the A and B serotype, as well as for E.

In total, the functional and immunological methods performed well, reaching similar percentages of success rate, i.e., correct positive and correct negative results taken together, and ranging from 90.2% to 98.1% for all serotypes except for immunological methods for BoNT/B which displayed 77.5% of the success rate (96.8% with functional methods). This lower value of the success rate obtained for BoNT/B is due to a combination of higher rates of false positive and lower rates of false negative results. Thus, the presence or absence of BoNT/B was more difficult to assess for some methods. Moreover, BoNT/B-spiked samples represented half of the sample set which also had an impact on the value of the success rate for the B serotype. The percentage of the success rate with immunological methods was high for BoNT/E and F, which were spiked at a medium concentration of 10 ng/mL, and which were of the same order of magnitude or better than the percentage of the success rate obtained with MBA, although this in vivo method was implemented by a lower number of participants (3/18). One should keep in mind that for BoNT/E and F serotypes, which represented only 2 samples out of 13, correct negative results had the greatest weight on the value of percentages of correct results. The dispersion of values of correct results for a given BoNT serotype was the highest with immunological methods giving percentages of success rate as low as 50% (Microarray-pBDi for BoNT/B) or reaching 100% (EL.R-1E for BoNT/E). This is consistent with immunological methods encompassing the highest diversity of tests involving antibodies with intrinsic differences in their performance of capture and detection of BoNTs, including subtype specificity. Only EL.R-1, an immunological method, covered all four BoNT serotypes with acceptable values of correct positive and correct negative results (95.2% to 100%). Within the group of functional methods, the MPN hemidiaphragm assay applied by one participant also gave excellent values of correct results (94.7% for BoNT/A to 100% for other BoNT serotypes). The standard mouse bioassay implemented by three participants performed similarly well for all serotypes with overall success rates between 92% and 94%, i.e., with a similar success rate to Endopep-MS for all four BoNT serotypes.

In conclusion, several methods, including different microarrays such as microarray bead in-house used by few participants for specific serotypes, gave excellent values of the success rate. Nevertheless, three methods were implemented by the highest number of participants, i.e., EL.R-1 (n = 7) and Endopep-MS (n = 5), for all BoNT serotypes, as well as LFA.CEA-Multi (n = 7) for BoNT/A, B, and E with good (66.4%/LFA.CEA-Multi) to excellent results comprised between 91.2% and 100% with EL.R1 and Endopep-MS. Among the group of functional methods implemented for all serotypes, the success rates were the highest with MPN, which was implemented by one participant only, followed by Endopep-MS and the MBA, the historic standard method of diagnostics.

### 2.4. Evaluation of Qualitative Results by Principle, Analyte, and Sample

Results were further analyzed according to methods grouped by principle and sample identity, e.g., comparison of results submitted for each given concentration of BoNT and matrix (Table 3).

Overall, the values of mean success rates were higher with functional methods than with immunological methods. For example, functional methods applied on the eight samples spiked with BoNT/B gave higher values of mean success rates of 100%, except for S18 (87.5%) and S10 (88.9%), as compared to immunological methods giving values of mean success rates which were comprised between 33.3% and 92.5%. This holds true for BoNT/A, for which greater values of mean success rates were obtained with functional methods except for S10 spiked with BoNT/B5 and BoNT/A4 produced by the bivalent strain Ba657 (Table 3). Of note, with the LC-MS/MS non-functional method deployed by two participants, the mean success rates reached 100% for all blank matrices and for the other selected samples with medium concentration (10 ng/mL) assessed, e.g., [S04 (BoNT/A1), S12 (BoNT/A1), S14 (BoNT/A3) and S18 (BoNT/B2)].

We noticed that the percentages of mean success rates with immunological methods were greater with samples spiked with BoNT/A than with BoNT/B. This most likely reflects the higher proportion of samples (*n* = 4) spiked at a low concentration of BoNT/B of 0.5 ng/mL versus one sample for BoNT/A (Table 3). Indeed, within the group of immunological methods, we recorded low percentages of success rates for BoNT/B spiked at 0.5 ng/mL (S15, S16, and S17) with no detected interference of the milk matrix (compare S15 with S17). Consistent with the absence of interference of the milk matrix, sample S07 provided as a blank matrix was correctly identified as a negative sample (100% mean success rate) independently of the method principle and serotype targeted. Contrasting with this, the serum-containing blank sample S03 turned out to be challenging for immunological methods giving low values of a mean success rate of 62.5% when targeting BoNT/A and 77.8% for BoNT/B.

With the sample S10 containing bivalent neurotoxins BoNT/B5 and BoNT/A4, we found higher percentages of the mean success rates for BoNT/B5 than for BoNT/A4, which is at least 1000-fold less active than BoNT/A1, especially with functional methods (35% mean success rates) for BoNT/A versus 88.9% mean success rates for BoNT/B. This indicates the overall influence of the neurotoxin subtype on methods of diagnosis of botulism. In line with this, we recorded similar values of the mean success rate for BoNT/A1 (S12) and BoNT/A3 (S14), spiked at identical concentrations in BSA/PBS with all three groups of methods. In contrast, we recorded differences in the mean success rates between BoNT/B1 (S08) and BoNT/B2 (S18) spiked at identical concentrations in either BSA/PBS or milk, with functional methods giving more homogenous results of 87.5% (S18) and 100% (S08) mean success rates.

Together these results show the impact of sample parameter variations on detection principles, including toxin concentrations, type of matrix, as well as variation of serotype and subtype.

### 2.5. Quantitative Results

Participants were asked to quantify BoNT/A and BoNT/B in the sample set. The recBoNT/A1 candidate reference material was provided to all participants to analyze the data with at least one standard calibration curve. Measurements reported by the participants were statistically evaluated according to the recommendations of the international standard ISO 13528:2015 [39]. More participants (10/17) reported quantitative results for BoNT/A than for BoNT/B (8/17).

ELISA methods were used for quantitative analyses of the PT samples by all participants together with candidate reference material for BoNT/A. Of note, the functional methods MPN and Endopep-MS were also applied by some of the participants. Some laboratories quantified only selected samples. The assigned values x_pt_ and their standard uncertainties were determined by the organizing laboratory in cooperation with ChemStat which calculated z’-scores for evaluation of the quantitative results. Details of statistical analyses are described in the Materials and Methods section.

**Figure 2:**
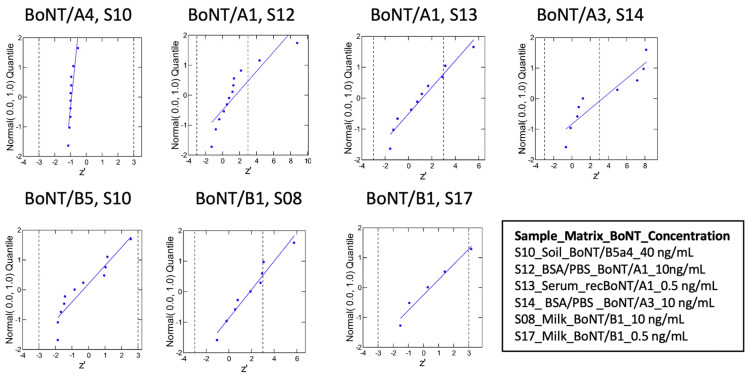
Normal probability plots of selected samples. The normal probability plots show z’-scores without extremes (z’ > 10), and without responses “<LoD” or “<LoQ” for samples with x_pt_ > 0.

Most laboratories reached acceptable z’-scores in the range of –3 to +3 for BoNT/A and BoNT/B. Examples of results provided in Figure 2 show z’-score variations between selected samples representing technical difficulties, i.e., spiked with bivalent toxin (S10) or low concentration of BoNT/A1 in serum, or to document the benefit of adding BoNT reference material. Data shown in Figure 2 show that laboratories that reported quantitative results for BoNT/A1 in serum (S13) provided z’-scores with a low level of dispersion. We recorded variations in z’-scores for S10 spiked with BoNT/B5 and BoNT/A4, showing less dispersion of values centered on −1 for BoNT/A4. This is most likely accounted for by the recBoNT/A1 candidate reference material provided to participants, thereby reaching its goal of reducing variations in quantitative analyses. Values of z’-scores for the BoNT/A3 rare subtype (S14, 10 ng/mL in 0.1% BSA/PBS) showed more deviation than data recorded with BoNT/A1 (S12, 10 ng/mL in 0.1% BSA/PBS). A toxin concentration effect was also observed when comparing dispersions of values between BoNT/B1 spiked in milk at 0.5 ng/mL (S17) and BoNT/B1 spiked in milk at 10 ng/mL (S08) (Figure 2).

Finally, several laboratories reached z’-scores above |3| for several more difficult samples (samples S04, S11, S13, S14, and S17). Moreover, six laboratories reached z’-scores above |10| for single different samples (=outliers that were excluded).

A simplified assessment is based on dichotomization of the quantitative results: the absolute differences D were compared to 3*σ*′p_t_ (see Section 4.2). Absolute differences smaller than this criterion are ‘acceptable’; otherwise, they are labeled as ‘unacceptable‘. Overall, the assessments showed that 90.4% of the reported results for BoNT/A were acceptable. These proportions were clearly better compared to a previous PT conducted in EuroBioTox (75.6%), in which more BoNT/A-containing samples were offered but candidate reference material recBoNT/A1 was not provided. For BoNT/B, 81.0% of the results were reported as acceptable even though no calibrant was supplied.

**Figure 3:**
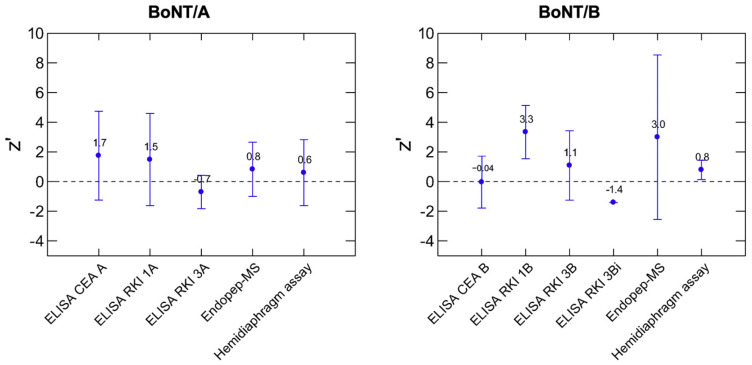
Overall z’-scores means for BoNT/A or BoNT/B quantification. The plots show the z’-score means (points) and their standard deviations (error bars span mean ± sd) as computed from the individual scores (results < LoD or <LoQ for BoNT-containing samples excluded).

The summarized z’-score means for BoNT/A and BoNT/B are depicted in the graph of Figure 3 together with the number of cases per method reported in Table S2. These data offer a guide to assess the mean closeness of a method to the assigned value if applied by several laboratories on several samples. The corresponding standard deviation shows the variation of the scores among the respective samples and laboratories. However, some of the methods have only very few data. Of note, the sandwich EL.R-1A giving the highest number of cases was obtained from seven laboratories.

The z’-scores in Figure 3 show a lower dispersion of values for BoNT/A than for BoNT/B, comprised between −0.7 and 1.7 for BoNT/A and −1.4 and 3.3 for BoNT/B. This difference is attributed to recBoNT/A1 candidate reference material provided to the participants, while different BoNT/B standards were used.

In conclusion, immunological methods EL.R-1A, EL.R-3A, EL.R-1B, EL.R-3B, EL.R-3Bi, e.g., EL.R-3B with different antibodies of capture and detection, and EL.C-A and EL.C-B when combined with recBoNT/A1 candidate reference material gave acceptable results as did the functional method Endopep-MS. Of note, the functional method MPN hemidiaphragm assay measuring the full biological activity yielded Z’ of 0.6 and 0.8 for BoNT/A and B, respectively, and thereby performed best above all methods. The quantitative results show that the quantification of BoNT/A, when compared to that of BoNT/B, was improved by providing reference material.

## 3. Conclusions and Perspectives

This PT organized within the EuroBioTox program targeted the recommended diagnostic methods for botulism including methods for which there was training during the EuroBioTox program. The data showed method dissemination between trained participants and efficacy, e.g., implemented by the greatest number of participants and giving the highest levels of correct results (both negative and positive) at low doses of BoNT, with blank matrices and on the broadest spectrum of BoNT serotypes spiked in complex matrices, including serum. Moreover, this PT established the importance of moving towards interlaboratory standardization of the procedures, by including training courses, and by providing reference material for standardized quantitative results. Collectively, these data provide groundbreaking information to decision-makers on the actual situation of botulism diagnostics in Europe and abroad as well as future directions for improving standardization of botulism data to be reported to the European Centre for Disease Prevention and Control, for example.

In total, the data showed excellent achievement by the large majority of participants with overall correct responses comprising between 63.8% and 98.9% and with 15 laboratories reaching total success rates greater than 80%. Despite the challenges that faced the analysis of a high number of BoNT subtypes (including a bivalent BoNT/A and B spiked sample), matrices, and low doses of BoNT, the qualitative data collected showed high rates (≥90%) of correct results for BoNT/A, E, and F with a slightly lower success rate of 83.7% for BoNT/B. This likely accounts for the high number of samples spiked with low concentrations of BoNT/B. Only two institutions reported results of BoNT sero- and subtypes by utilizing mass spectrometry methods in the bonus task. Both laboratories reported correct results for the four targeted samples (S07, S12, S14, and S18). There were two correct results without subtyping and one false result for sample S18, which was a rather challenging sample due to the medium concentration of spiked toxin and potentially complex subtype (BoNT/B2). One laboratory reported a partially false result for the sample S14 spiked with BoNT/A3. Collectively, these results highlight the importance of moving forward with methods that target equally well all BoNT subtypes commonly responsible for human botulism.

Half of the participants successfully implemented one or more trained immunological methods developed by RKI and CEA thanks to material rendered accessible on the EuroBioTox repository with a targeted success greater than 95% for BoNT/A. In addition, the antibodies available in the EuroBioTox repository were successfully used by the participants for the detection of BoNT/B. Inspection of the respective standard curves showed that the sensitivity of trained methods implemented by participants can be further improved, calling for the importance of pursuing method training.

In this PT, immunological methods were employed twice more than functional methods and about 20 times more than the non-functional LC-MS/MS method. The LC-MS/MS method showed excellent results for blank samples and samples spiked at medium concentration of BoNTs, pointing to the interest in demonstrating its efficacy at low doses of the toxin. Immunological methods showed the highest diversity with no less than 18 ELISA-based methods and 6 LFA-based methods. Of note, 10 participants implemented several technical approaches and reported results based on the different techniques, while the other eight laboratories used preferentially one technology (e.g., either ELISA or MBA). These differences in the implementation of techniques should be considered when comparing the success rates per participant and method.

In conclusion, the results provided in this proficiency test represent valuable information regarding the good level of preparedness of laboratories specialized in the diagnostics of botulism in Europe and abroad as well as the remarkable dynamics in developing analytical methods in the field. It is important to strengthen the use of *in vitro* methods of diagnosis to reduce the use of MBA, improve sensitivity, and provide quantitative information. Combining methods based on different principles, including standard methods and reference material, represents a way both to deal with difficult samples and report standardized quantitative data at a European level.

## 4. Materials and Methods

### 4.1. Materials

Under the EuroBioTox program, large batches of recombinant BoNT/A1 (recBoNT/A1) and recBoNTB1 were produced to establish certified reference material (EURM^®^-111, recBoNT/A1; EURM^®^-112 recBoNT/B1), here referred to as candidate reference material (see accompanying article in the Special Issue). Recombinantly expressed botulinum toxins recBoNT were purified, as previously described [40]. The recBoNT/E1 and recBoNT/F1 used to defined assigned concentrations of BoNT/E1 and BoNT/F1 were produced and purified as reported for recBoNT/A1 and provided by Toxogen [40]. Native BoNT BoNT/A1 producing strain Hall, BoNT/A3 producing strain Loch Maree, BoNT/B1 producing strain 7273, BoNT B2 producing strain BL6, BoNTB5a4 producing strain Ba657, BoNT/E1 producing strain 9009, and BoNT/F1 producing strain NCIB 10658 were semi-purified, as previously described [41]. The concentrations of native toxin complexes were estimated by the sandwich ELISA with the combination of capture and detection antibodies reported in Table S3 and by using recombinant recBoNT/A1, B1, E1, and F1 as standards. BoNTs were spiked either in PBS 0.1% BSA/PBS (Sigma-Aldrich, Munich, Germany) or in complex food, clinical, and environmental matrices to prepare the PT samples. As a complex food matrix, we used 5% ultra-high temperature (UHT) semi-skimmed milk purchased from a local retail store. Pooled human serum (SIGMA) and soil (100% fine-grained < 1.0 mm washed natural sand) were selected as typical clinical and environmental matrices.

### 4.2. Methods

The preparation and conduct of the proficiency test on botulinum neurotoxins have been detailed [36] and are briefly described below. Neurotoxins were either recombinantly expressed in *Escherichia coli* and purified (recBoNT) [40] or semi-purified from *C. botulinum* culture supernatants as native progenitor complexes (BoNT), as previously described [41]. The additional steps of centrifugation and filtration of semi-purified native PTC samples for sterilization were conducted.

#### 4.2.1. Stability Test

Before the selection of the sample set, we analyzed the samples for stability according to the ISO 13528:2015. Indeed, samples had to be sufficiently stable during the predefined testing period which was set to 7 weeks. Stability testing was performed and analyzed by the sandwich ELISA (RKI), specifically detecting BoNT/A, B, E, and F (Table S3). A total of 10 aliquots of each sample were prepared and analyzed to demonstrate sample stability during the PT test period. To this aim, five aliquots were stored at −80 °C for four weeks (reference temperature), and five aliquots were stored at +4 °C for seven weeks (storage condition for the samples during the PT). Finally, we conducted a post-stability test on PT samples.

A material was considered sufficiently stable if the absolute value of the difference between samples stored at +4 °C and −80 °C was ≤0.3 × *σ_pt_*. Briefly, the data showed that BoNT/A and BoNT/E were sufficiently stable. Though some increasing values recorded for BoNT/B did not meet the ISO criterion, these samples were kept for the proficiency test, except for S09 containing unstable BoNT/B. Indeed, we considered that the increase of concentration measured likely reflected poor intra-laboratory ELISA reproducibility or was due to evaporation of the solvent during the 7 weeks of storage at 4 °C. In total, we found insufficient stability for samples S05 and S09, which were removed from the exercise, while we kept S10 to assess the capabilities of participants to detect bivalent BoNT/B5a4. Only samples spiked with BoNT/A or BoNT/B were selected for quantitative analysis in the proficiency test.

#### 4.2.2. Homogeneity Test

For the actual proficiency test, 40 aliquots of each of the selected 16 samples had been prepared. Of the 13 BoNT-spiked samples, 10 aliquots of each sample were randomly selected for homogeneity testing. The homogeneity of each test material was assessed according to ISO 13528:2015 by employing the corresponding sandwich ELISA and deducing the concentration of the 10 test portions of each sample. Each sample was quantified twice in duplicate by ELISA (Table S4).

The assigned values of BoNT in samples, as opposed to theoretically spiked concentrations, were estimated as follows. For each randomly selected aliquot, one dilution close to the EC_50_ value of the corresponding ELISA for BoNT/A, B, E, and F was chosen to calculate the toxin concentrations. The dilutions were measured in duplicate in two independent experiments. The experimental data obtained in the homogeneity testing for the 10 randomly selected aliquots were used to determine the concentrations of the PT samples. The calculated concentrations of the ten replicates of each sample measured in two experiments were statistically analyzed, and estimates of the mean concentrations were obtained according to ISO 13528:2015, C.3. These robust means of toxin concentrations were adopted as “assigned values”, *x_pt_*, for the evaluation of the quantitative results reported by the participants in this proficiency test on BoNT/A and BoNT/B (Table 1). In addition, for the subsequent quantitative analysis of proficiency test results reported by the participants, the standard deviation for proficiency assessment, *σ_pt_*, was calculated as 0.25·*x_pt_*.

#### 4.2.3. General Considerations of Methods Implemented by the Participants

The wide range of methods used by the participants is listed in Table S1 with each method’s attributed ID used for evaluation. Different functional, immunological, and mass spectrometry-based methods were deployed in this proficiency test. This encompassed (i) *in vivo* MBA that detected a few pg of all BoNTs pathogenic to humans and (ii) the *ex vivo* MPN or HDA [18,19]. Other functional methods detected the endopeptidase activities of LCs on SNARE proteins with serotype specificities. These methods are coupled to different types of read-outs, such as the mass spectrometry-based method of detection (Endopep-MS) [26] or immunological detection of cleavage products (Endopep-ELISA) [27,28]. These *in vitro* methods aimed at detecting BoNT enzymatic activities encompass a step of immunoaffinity enrichment, where the toxin is captured from the matrix using antibody-coated microbeads, thereby resulting in assay sensitivities similar or even better than MBA [14,29]. In endopeptidase assays the HC is the preferred target of antibodies, or receptor-binding HCC, for toxin extraction from the sample [42]. Non-functional methods, including immunological methods, have been developed to quantify toxin amounts. This encompasses the sandwich ELISA, which is by far the most employed *in vitro* method to detect BoNT and quantify the amount of the toxin [14,20,21,22,23]. These methods provide valuable information on serotypes but do not allow subtyping. Depending on the quality of the antibodies, these robust methods can reach high sensitivity for multiple samples in a short period of analysis. This holds for immunochromatographic lateral flow assays, which are hand-held devices based on a sandwich ELISA performed on paper strips. Usually, they have lower sensitivity compared to the conventional ELISA and do not provide quantitative results also being frequently the subject of matrix interferences [14]. The development of methods aimed at miniaturizing the detection of BoNT or BoNT endopeptidase activities have been reported and encompass microarrays or biosensors, centrifugal microfluidic disk platforms, and portable devices [32,33,34,35].

The data in the qualitative analysis report sheet were reported by a code (0, 1, and na) according to the categories (absent, present, not analyzed/not available). The participants were asked to indicate the limit of detection if it was known in the same units as the result (ng/mL). Quantitative results were reported for the samples in ng/mL. The participants were requested to insert 0 (zero), if results were below the limit of detection (LoD), or limit of quantification (LoQ). The submitted data were statistically analyzed according to the procedures defined in ISO 13528:2015. The z-scores were calculated and reported only if the assigned values of the concentrations and the ‘standard deviations for proficiency assessment’ could be identified with a sufficient degree of reliability.

#### 4.2.4. Statistical Analysis and Data Visualization

Data obtained in the pre- and post-PT stability studies as well as from the homogeneity study were statistically evaluated and assessed according to the procedures and criteria recommended by ISO 13528:2015. Outlier tests (Grubbs, Cochran) were conducted using R (R version 4.1.0) and R-package outliers (Package outliers version 0.14) to identify extreme values. Stability data were assessed according to equation B.17 of this standard, $\left|{\bar{y}}_{1}-{\bar{y}}_{2}\right|\leq 0.3\sigma_{pt}$ i.e., the difference of means at different storage conditions was compared to $0.3\sigma_{pt}$. The assessment of between-unit homogeneity followed the recommendations and statistical procedures in Ch. 6.1 and Annex B; assigned values were calculated from the data of the homogeneity study according to Annex C.3, Algorithm A, using the R-package metRology (metRology version 0.9-28-1).

Qualitative responses (categories “0”, “1” and “n.a.”) were compared to the correct answers by simple algorithms coding auxiliary variables representing “success”: 1 for correct positive, −1 for correct negative, 10 for false positive, and −10 for false negative. The latter coding of single responses led to mean values of 4.5 and −4.5 if one of the two reported results was false positive or false negative, respectively. For dichotomic grouping (correct/false) it was sufficient to use codes 1 and 0, respectively. Based on these categorical auxiliary variables, the success rates were obtained by frequency tabulation or by computing the means of the (0/1)-coded auxiliary variables grouped by the categories of interest (e.g., grouped by method).

Quantitative PT results are as follows. The quantitative data reported by the participants were statistically evaluated according to ISO 13528:2015, chapter 9:

z-scores, differences *D*, and relative differences *D* [%], are defined as follows:

$$z=\frac{x-x_{pt}}{\sigma_{pt}};\;\;D=x-x_{pt};\;\;\;D\;[\%]=100\cdot \frac{x-x_{pt}}{x_{pt}}\;[\%]$$

where *x* is the reported quantitative result, *x_pt_* the assigned value defined by the organizer, and *σ_pt_* standard deviation for the proficiency assessment (*σ_pt_* was set to 0.25·*x_pt_*).

The conventional interpretation of z scores is as follows:

- A result that gives |*z*| ≤ 2.0 is considered to be acceptable.
- A result that gives 2.0 < |*z*| < 3.0 is considered to give a warning signal.
- A result that gives |*z*| ≥ 3.0 is considered to be unacceptable (or action signal).

z’ scores are defined in ISO 13528:2015, Equation (15):

$$z^{\prime }=\frac{x-x_{pt}}{\sqrt{\sigma_{pt}^{2}+u^{2}(x_{pt})}}$$

The squared denominator is enlarged by the squared uncertainty of the assigned value, $u^{2}(x_{pt})$. In the present case, this additional term covers the squared standard uncertainty of the assigned values as computed from the homogeneity data and the squared difference between the values *x_ps_* observed *post*-PT by the organizing laboratory, and the assigned values; i.e., the differences are treated as variance components:

$$z^{\prime }=\frac{x-x_{pt}}{{\sigma^{\prime }}_{pt}},\;\;\mathrm{with}\;{{\sigma^{\prime }}_{pt}}^{2}=\sigma_{pt}^{2}+u^{2}(x_{pt})+{\left|x_{ps}-x_{pt}\right|}^{2}.$$

*D* and *D* [%] scores were compared with *d_E’_*; where *d_E’_* was set to 3*s’_pt_*. If –*d_E’_* < *D* < *d_E’_*, then the performance was considered to be ‘acceptable’.

Normal probability plots of the z’-scores were produced to visualize the empirical distributions of the results reported, as compared to the model implicitly set as a normal distribution with mean *x_pt_* and variance $\sigma_{pt}^{2}$. Assessment of the accordance of methods was based on the arithmetic means of the individual z’-scores. Statistics and graphs of the quantitative results were produced with SYSTAT 13.0 (Systat Software, Inc., Chicago, MI, USA).

## Figures and Tables

**Table 1:** List of selected samples with theoretical and assigned concentrations. Of note, recBoNT stands for recombinantly produced and purified BoNT. Native BoNT was partially purified from bacterial culture supernatants. Theoretical concentrations were calculated according to values defined for the stock solution. Assigned values were determined by ELISA for each sample and show differences as compared to theoretical concentrations that can be attributed to losses during sample preparation, matrix interference effects, or other disturbing factors.

Sample	Matrix	Spiked Toxin	Assigned Concentration [ng/mL]	Theoretical Concentration [ng/mL]
S01	Soil	–	–	–
S02	0.1% BSA/PBS	BoNT/F1	5.31	10
S03	Serum	–	–	–
S04	Milk	recBoNT/B1	6.62	10
S06	0.1% BSA/PBS	BoNT/E1	0.59	10
S07	Milk	–	–	–
S08	Milk	BoNT/B1	1.98	10
S10	Soil	BoNT/B5a4	38.74 (B5) 18.93 (A4)	40
S11	0.1% BSA/PBS	recBoNT/B1	0.66	0.5
S12	0.1% BSA/PBS	BoNT/A1	11.97	10
S13	Serum	recBoNT/A1	0.64	0.5
S14	0.1% BSA/PBS	BoNT/A3	17.45	10
S15	0.1% BSA/PBS	BoNT/B1	0.15	0.5
S16	Milk	recBoNT/B1	0.38	0.5
S17	Milk	BoNT/B1	0.12	0.5
S18	0.1% BSA/PBS	BoNT/B2	1.31	10

**Table 2:** Unconditional success rates for a given method and BoNT serotype. For each given method or group of methods and BoNT serotype, the table shows the number of results (total counts) correctly reported as positive (correct positive) or negative (correct negative) divided by the total number of results reported as either correct or false and expressed as percentages [%]. Data summary by type of method appear with a blue color background.

METHODS	BoNT/A				BoNT/B				BoNT/E				BoNT/F			
	Total Count	Correct Positive [%]	Correct Negative [%]	Correct [%]	Total Count	Correct Positive [%]	Correct Negative [%]	Correct [%]	Total Count	Correct Positive [%]	Correct Negative [%]	Correct [%]	Total Count	Correct Positive [%]	Correct Negative [%]	Correct [%]
ALL METHODS	901	21.5	69.6	91.1	849	37.1	46.6	83.7	640	5.5	90.6	96.1	389	5.1	91.8	96.9
FUNCTIONAL METHODS	286	21.7	71	92.7	256	47.6	49.2	96.8	229	3.9	91.7	95.6	223	5	91	96
Endopep-ELISA Bead A+B	16	25	75	100	16	50	50	100	-	-	-	-	-	-	-	-
Endopep-MS	181	21	70.2	91.2	145	50.3	48.3	98.6	144	4.9	91	95.9	144	6.3	90.3	96.6
Hemidiaphragm assay (HDA or MPN)	19	21.1	73.7	94.8	19	36.8	63.2	100	19	0	100	100	19	10.5	89.5	100
Mouse Bioassay (MBA)	70	22.9	71.4	94.3	76	44.7	47.4	92.1	66	3	90.9	93.9	60	0	93.3	93.3
IMMUNOLOGICAL METHODS	603	21.2	69	90.2	576	32.7	44.8	77.5	399	6.5	89.8	96.3	154	5.9	92.2	98.1
ELISA CEA A, B or E	46	30.4	65.2	95.6	54	51.9	33.3	85.2	32	6.3	93.8	100	-	-	-	-
BioSentinel E	-	-	-	-	-	-	-	-	8	0	75	75	-	-	-	-
ELISA in-house 2A or 2B	32	12.5	68.8	81.3	32	37.5	37.5	75	-	-	-	-	-	-	-	-
ELISA RKI 1A, 1B, 1E or 1F	201	27.9	70.6	98.5	104	49	46.2	95.2	112	6.3	93.8	100	32	6.3	90.6	96.9
ELISA RKI 2F or 2E	-	-	-	-	-	-	-	-	2	100	-	100	2	100	-	100
ELISA RKI 3A, 3B or 3F	32	15.6	53.1	68.7	70	42.9	45.7	88.6	-	-	-	-	32	-	93.8	93.8
ELISA RKI 3Bi	-	-	-	-	32	12.5	50	62.5	-	-	-	-	-	-	-	-
ELISA RKI 4F	-	-	-	-	-	-	-	-	-	-	-	-	32	6.3	93.8	100
LFA CEA Multiplex	196	15.3	71.9	87.2	196	18.4	48	66.4	196	6.1	87.8	93.9	-	-	-	-
LFA Miprolab A or B	40	17.5	65	82.5	32	37.5	43.8	81.3	-	-	-	-	-	-	-	-
Microarray (ELISA-based) pBDi	8	0	62.5	62.5	8	0	50	50	1	0	0	0	8	0	100	100
Microarray (ELISA-based) Bead commercial	32	25	65.6	90.6	32	25	37.5	62.5	32	6.3	93.8	100	32	6.3	93.8	100
Microarray (ELISA-based) Bead in-house	16	25	75	100	16	43.8	50	93.8	16	6.3	93.8	100	16	6.3	93.8	100
LC-MS/MS	12	33.3	66.7	100	17	29.4	70.6	100	12	0	100	100	12	0	100	100
LC-MS/MS	12	33.3	66.7	100	17	29.4	70.6	100	12	0	100	100	12	0	100	100

**Table 3:** Qualitative results are grouped by analytical principles, samples S1 to S18, and analytes BoNT/A, B, E, and F. To facilitate reading, the composition of each sample is given in parentheses (matrix, BoNT serotype, theoretical concentration in ng/mL; with O: soil, M: milk, P: BSA/PBS). Mean success rates per analytical principle and sample are computed by the proportion of correct results, including correct negative and correct positive results. Each correct result was assigned a one (1), each false result a zero (0), and then the grouped means of these assignments gave the proportions, which were finally multiplied by 100 to give the mean percentages [%].

Principles	Sample	BoNT/A		BoNT/B		BoNT/E		BoNT/F	
		n	Mean Success Rate [%]	n	Mean Success Rate [%]	n	Mean Success Rate [%]	n	Mean Success Rate [%]
functional methods	S01 (O)	20	90.0	18	88.9	17	88.2	15	100.0
	S02 (P_F1_10)	16	100.0	14	85.7	13	100.0	13	84.6
	S03 (R)	19	89.5	18	100.0	15	100.0	15	100.0
	S04 (M_rB1_10)	18	100.0	16	100.0	15	100.0	15	86.7
	S06 (P_E1_10)	16	100.0	14	100.0	15	60.0	13	76.9
	S07 (M)	20	100.0	18	100.0	17	100.0	15	100.0
	S08 (M_B1_10)	17	100.0	15	100.0	14	100.0	14	85.7
	S10 (R_B5a4_40)	20	35.0	18	88.9	14	85.7	14	100.0
	S11 (P_rB1_10)	17	100.0	15	100.0	14	100.0	14	100.0
	S12 (P_A1_10)	19	100.0	16	100.0	15	100.0	15	100.0
	S13 (R_rA1_0.5)	19	89.5	16	100.0	13	100.0	13	100.0
	S14 (P_A3_10)	19	100.0	16	100.0	15	100.0	15	100.0
	S15 (P_B1_0.5)	16	100.0	16	100.0	13	100.0	13	100.0
	S16 (M_rB1_0.5)	18	88.9	16	100.0	13	100.0	13	100.0
	S17 (M_B1_0.5)	16	100.0	14	100.0	13	100.0	13	100.0
	S18 (P_B2_10)	16	100.0	16	87.5	13	100.0	13	100.0
immunological methods	S01 (O)	37	94.6	35	100.0	26	100.0	9	100.0
	S02 (P_F1_10)	35	94.3	33	100.0	24	100.0	11	81.8
	S03 (R)	40	62.5	36	77.8	24	83.3	10	100.0
	S04 (M_rB1_10)	37	100.0	39	92.3	24	100.0	10	100.0
	S06 (P_E1_10)	37	100.0	34	100.0	29	89.7	10	100.0
	S07 (M)	35	100.0	33	100.0	24	100.0	9	100.0
	S08 (M_B1_10)	38	100.0	40	92.5	25	100.0	10	100.0
	S10 (R_B5a4_40)	41	61.0	38	76.3	27	100.0	10	100.0
	S11 (P_rB1_10)	35	97.1	38	68.4	24	95.8	9	100.0
	S12 (P_A1_10)	43	86.0	35	100.0	25	100.0	10	100.0
	S13 (R_rA1_0.5)	38	76.3	36	72.2	24	75.0	10	100.0
	S14 (P_A3_10)	42	88.1	34	100.0	25	96.0	9	100.0
	S15 (P_B1_0.5)	37	97.3	36	33.3	24	100.0	9	100.0
	S16 (M_rB1_0.5)	37	100.0	36	50.0	24	100.0	9	88.9
	S17 (M_B1_0.5)	35	97.1	36	33.3	26	100.0	9	100.0
	S18 (P_B2_10)	36	97.2	37	48.6	24	100.0	10	100.0
mass spectrometric methods (not functional)	S01 (O)	2	100.0	2	100.0	2	100.0	2	100.0
	S02 (P_F1_10)	-	-	-	-	-	-	-	-
	S03 (R)	2	100.0	2	100.0	2	100.0	2	100.0
	S04 (M_rB1_10)	-	-	1	100.0	-	-	-	-
	S06 (P_E1_10)	-	-	-	-	-	-	-	-
	S07 (M)	2	100.0	3	100.0	2	100.0	2	100.0
	S08 (M_B1_10)	-	-	-	-	-	-	-	-
	S10 (R_B5a4_40)	-	-	1	100.0	-	-	-	-
	S11 (P_rB1_10)	-	-	-	-	-	-	-	-
	S12 (P_A1_10)	2	100.0	2	100.0	2	100.0	2	100.0
	S13 (R_rA1_0.5)	-	-	-	-	-	-	-	-
	S14 (P_A3_10)	2	100.0	3	100.0	2	100.0	2	100.0
	S15 (P_B1_0.5)	-	-	-	-	-	-	-	-
	S16 (M_rB1_0.5)	-	-	-	-	-	-	-	-
	S17 (M_B1_0.5)	-	-	-	-	-	-	-	-
	S18 (P_B2_10)	2	100.0	3	100.0	2	100.0	2	100.0

## Data Availability

The original contributions presented in the study are included in the article/Supplementary Material, further inquiries can be directed to the corresponding author.

## Supplementary Materials

The following supporting information can be downloaded at: https://www.mdpi.com/article/10.3390/toxins16110485/s1, Table S1: Methods by ID used by the participants. This table summarizes the principles and combinations of methods with their respective antibodies used to detect BoNT according to the acronym given (Abbreviation). (*) Methods selected for training during EuroBioTox and before this proficiency test. Of note, while EL.R-1A was the method selected for training, both the antibodies used for EL.R-1A and 1B were available in the EuroBioTox repository; Table S2: Number of cases obtained by the method. Note that the amount of data per method (n) shows a high dispersion of values, which has an impact on the mean and standard deviation values; Table S3: List of monoclonal and polyclonal antibodies used for sandwich ELISA provided by RKI. Monoclonal (mAb) and polyclonal (pAb) antibodies were used to detect BoNT/A, B, E, and F to characterize the PT samples; Table S4: Summary statistics of homogeneity data. Data were processed according to ISO 13528:2015, B.2.2 (a) and ISO 13528:2015, B.2.3 (b). ss: between-unit standard deviation (sampling standard deviation); σp: Standard deviation for proficiency assessment; sr: repeatability standard deviation; σall: "Allowed" sampling standard deviation.
