# Supplementary material for: International Proficiency Test Targeting a Large Panel of Botulinum Neurotoxin Sero- and Subtypes in Different Matrices
Source: Toxins (Basel). 2024 Nov 8;16(11):485. doi: 10.3390/toxins16110485 (PMC11598462; doi:10.3390/toxins16110485)
Supplement: Supplementary file 1 [file toxins-16-00485-s001.zip › toxins-3283609-supplementary.pdf]

**Supplementary Material**

**Supplementary Table S1.** Methods by ID used by the participants. This table summarizes the principles and combinations of methods with their respective antibodies used to detect BoNT according to the acronym given (Abbreviation). (*) Methods selected for training during EuroBioTox and before this proficiency test. Of note, while EL.R-1A was the method selected for training, both the antibodies used for EL.R-1A and 1B were available in the EuroBioTox repository.

| Method | Toxin | Assignment | Abbreviation (Method ID) |
|---|---|---|---|
| ELISA commercial | BoNT/E | ELISA BioSentinel E | EL.BioSen-E |
| ELISA RKI | BoNT/A | ELISA RKI 1A* | EL.R-1A |
| | | ELISA RKI 2A | EL.R-2A |
| | | ELISA RKI 3A | EL.R-3A |
| | BoNT/B | ELISA RKI 1B | EL.R-1B |
| | | ELISA RKI 3B | EL.R-3B |
| | BoNT/E | ELISA RKI 1E | EL.R-1E |
| | | ELISA RKI 2E | EL.R-2E |
| | BoNT/F | ELISA RKI 1K | EL.R-1F |
| | | ELISA RKI 2F | EL.R-2F |
| | | ELISA RKI 3F | EL.R-3F |
| | BoNT/A | ELISA in-house 2A | EL.2-A |
| | BoNT/B | ELISA in-house 2B | EL.2-B |
| | BoNT/E | ELISA in-house 2E | EL.2-E |
| | BoNT/A, B, E | ELISA in-house A + B + E | EL.-A/B/E |
| ELISA CEA | BoNT/A | ELISA CEA A* | EL.C-A |
| | BoNT/B | ELISA CEA B | EL.C-B |
| | BoNT/E | ELISA CEA E | EL.C-E |
| LFA commercial | BoNT/A, B, E | LFA CEA Multiplex | LFA.CEA-Multi |
| | BoNT/A | LFA Miprolab A | LFA.Mipro-A |
| | BoNT/B | LFA Miprolab B | LFA.Mipro-B |
| | BoNT/A, B | LFA Bot Tox,Tetracore | LFA.Tetra |
| | BoNT | LFA RAID Tox, Alexeter | LFA.Alex |
| | | LFA ProStrips, Advnt | LFA.Advnt |
| Endopep-ELISA | BoNT/A, B | Endopep-ELISA Bead A + B | EpEL-A/B |
| LC-MS/MS | BoNT/A | MS/MS (LC) | MS/MS |
| | BoNT/B | MS/MS (LC) | MS/MS |
| | BoNT/E | MS/MS (LC) | MS/MS |
| | BoNT/F | MS/MS (LC) | MS/MS |
| LC-MS/MS (HR) | BoNT/A-F | MS/MS (LC) | MS/MS |
| Endopep-MS | BoNT/A | Endopep-MS | EpMS |
| | BoNT/B | Endopep-MS | EpMS |
| | BoNT/E | Endopep-MS | EpMS |
| | BoNT/F | Endopep-MS | EpMS |
| Mouse Bio Assay | BoNT, A, B, E, F | Mouse bioassay | MBA |
| Microarray (ELISA-based) | BoNT/A-F | Microarray (ELISA-based) Bead in-house | MAR.1 |
| | BoNT/A-F | Microarray (ELISA-based) pBDi | MAR.2 |
| | BoNT/A-F | Microarray (ELISA-based) Bead commercial | MAR.3 |
| Hemidiaphragm assay | BoNT, A-F | Hemidiaphragm assay | HDA or MPN |

**Supplementary Table S2.** Number of cases obtained by the method. Note that the amount of data per method (n) shows a high dispersion of values, which has an impact on the mean and standard deviation values.

| Measurand | Method | n |
|---|---|---|
| BoNT/A | ELISA CEA A | 6 |
| | ELISA RKI 1A | 26 |
| | ELISA RKI 3A | 3 |
| | Endopep-MS | 3 |
| | Hemidiaphragm assay | 3 |
| BoNT/B | ELISA CEA B | 14 |
| | ELISA RKI 1B | 21 |
| | ELISA RKI 3B | 16 |
| | ELISA RKI 3Bi | 1 |
| | Endopep-MS | 3 |
| | Hemidiaphragm assay | 4 |

**Supplementary Table S3.** List of monoclonal and polyclonal antibodies used for sandwich ELISA provided by RKI. Monoclonal (mAb) and polyclonal (pAb) antibodies were used to detect BoNT/A, B, E, and F to characterize the PT samples.

| **BoNT serotypes** | **Coating antibody** | **Detection antibody** |
|---|---|---|
| BoNT/A | mAb A778 + mAb A2807 | mAb A120-Bio |
| BoNT/B | mAb B279 | polyclonal horse antiserum Behring-Bio (Novartis) |
| BoNT/E | pAb KE97 | mAb E1447-Bio |
| BoNT/F | mAb F189 | pAb HF59 |

**Supplementary Table S4.** Summary statistics of homogeneity data. Data were processed according to ISO 13528:2015, B.2.2 (a) and ISO 13528:2015, B.2.3 (b). $s_s$: between-unit standard deviation (sampling standard deviation); $\sigma_p$: Standard deviation for proficiency assessment; $s_r$: repeatability standard deviation; $\sigma_{all}$: "Allowed" sampling standard deviation.

| **Measurand** | **Material** | **Assigned value** | $s_s$ | $\sigma_{pt}$ | $s_r$ | $\sigma_{all}$ | **unit** | $s_s/\sigma_{pt}$ (a) | $s_r/\sigma_{pt}$ | **Conclusion** (b) |
|---|---|---|---|---|---|---|---|---|---|---|
| BoNT/A4 | S10 | 38.7 | 1.76 | 9.69 | 1.24 | 2.91 | ng/mL | 0.18 | 0.13 | homogeneity sufficient |
| BoNT/A1 | S12 | 12.0 | 0.921 | 2.99 | 0.796 | 0.898 | ng/mL | 0.31 | 0.27 | homogeneity sufficient |
| BoNT/A1 | S13 | 0.646 | 0.0414 | 0.162 | 0.0591 | 0.0485 | ng/mL | 0.26 | 0.37 | homogeneity sufficient |
| BoNT/A3 | S14 | 17.4 | 0.998 | 4.36 | 1.42 | 1.31 | ng/mL | 0.23 | 0.33 | homogeneity sufficient |
| BoNT/B1 | S04 | 6.62 | 0.263 | 1.66 | 0.743 | 0.497 | ng/mL | 0.16 | 0.45 | homogeneity sufficient |
| BoNT/B1 | S08 | 1.99 | 0.113 | 0.497 | 0.170 | 0.149 | ng/mL | 0.23 | 0.34 | homogeneity sufficient |
| BoNT/B5 | S10 | 18.9 | 0.0818 | 4.73 | 1.25 | 1.42 | ng/mL | 0.017 | 0.26 | homogeneity sufficient |
| BoNT/B1 | S11 | 0.662 | 0.0538 | 0.165 | 0.0735 | 0.0496 | ng/mL | 0.33 | 0.44 | homogeneity sufficient |
| BoNT/B1 | S15 | 0.153 | 0.0114 | 0.0382 | 0.00646 | 0.0114 | ng/mL | 0.30 | 0.17 | homogeneity sufficient |
| BoNT/B1 | S16 | 0.390 | 0.0201 | 0.0975 | 0.0250 | 0.0292 | ng/mL | 0.21 | 0.26 | homogeneity sufficient |
| BoNT/B1 | S17 | 0.128 | 0.00667 | 0.0321 | 0.0105 | 0.00964 | ng/mL | 0.21 | 0.33 | homogeneity sufficient |
| BoNT/B2 | S18 | 1.32 | 0.0755 | 0.329 | 0.0990 | 0.0987 | ng/mL | 0.23 | 0.30 | homogeneity sufficient |
| BoNT/E1 | S06 | 0.593 | 0.0184 | 0.148 | 0.0803 | 0.0445 | ng/mL | 0.12 | 0.54 | homogeneity sufficient |
| BoNT/F1 | S02 | 5.32 | 0.508 | 1.33 | 0.627 | 0.399 | ng/mL | 0.38 | 0.47 | homogeneity sufficient |
